# The efficacy of disinfectants on abattoirs’ *Candida albicans* isolates in Niger Delta region

**DOI:** 10.12688/f1000research.1-20.v1

**Published:** 2012-10-01

**Authors:** Oluwayemisi A Olorode, G Chijioke Okpokwasli

**Affiliations:** 1 Department of Pharmaceutical Microbiology and Biotechnology, Faculty of Pharmacy, Niger Delta University, Wilberforce lsland, Bayelsa State, Nigeria; 2 Department of Microbiology, Faculty of Science, University of Port Harcourt, Rivers State, Nigeria

## Abstract

This study was conducted to evaluate the antimicrobial activities of common disinfectants- these are (parachlorometaxylenol) dettol, savlon purit and jik (sodium hypochlorite) on *Candida albicans* isolated from displaying and cutting tables in five different abattoirs in Port Harcourt (Niger Delta region); the abattoirs include Trans Amadi, Agip, Woji, Rumuokoro, and Rumuodara. This research was carried out between January 2005 and June 2006. Swab samples were collected from abattoirs cutting tables with sterile swab sticks and immediately transferred and cultured in the laboratory on a selective medium Sabouraud Dextrose Agar (SDA). The disinfectants’ concentrations were prepared at 10%, 20%, 40%, and 70%, in triplicates and the mean values calculated. 0.5 Mc Farland turbidity method of standardization and Agar diffusion method were used for disinfectants testing of the isolates. Statistical analysis of the data showed no significant difference in the effectiveness of these disinfectants at (p<0.05). In conclusion, this study has shown that savlon and dettol were the most potent antimicrobial agents at 10% concentration on *Candida albicans* isolates when compared with purit and jik in this study, hence they are good sanitizing agents to be applied on the abattoirs cutting tables, before meat products can be displayed for sale.

## Introduction

*Candida albicans* is a diploid fungus that grows both as yeast and filamentous cells and a causal agent of opportunistic oral and genital infections in human ^1, 2^. The yeast *Candida albicans* is an opportunist fungal pathogen which can be present as a normal part of body’s microflora ^3^. *Candida albicans* displays a variety of virulence factors which aid colonization and persistence in the body. One of the most important of these factors is the ability to adhere to host tissue using a variety of mechanisms. The importance of adherence may be illustrated by the ability of *Candida albicans* to adhere to various mucosal surfaces and to withstand forces that may lead to its removal from the body such as the bathing/washing action of body fluids. A hierarchy exists among Candida species indicating that the more common aetiological agents of candidiosis ( *C. albicans* and *C. tropicalis*) are more adherent to host tissues *in vitro* than relatively non-pathogenic species such as *C. krusei* and *C. guilliermondii* ^4^. Systemic fungal infections (fungemias) including those by *Candida albicans* have emerged as important causes of morbidity and mortality in immunocompromised patients (e.g. AIDS, cancer chemotherapy, organ or bone marrow transplantation). *Candida albicans* biofilms may form on the surface of implantable medical devices. In addition, hospital-acquired infections by *Candida albicans* have become a cause of major health concerns.

Calderone (2002) stated that *Candida albicans* is commensal and a constituent of the normal gut flora comprising microorganisms that live in the human mouth and gastrointestinal tract. *C. albicans* lives in 80% of the human population without causing harmful effects, although overgrowth of the fungus results in candidiasis (candidosis) ^5^. Candidiasis is often observed in immunocompromised individuals such as HIV-infected patients. A common form of candidiasis restricted to the mucosal membranes in mouth or vagina is thrush, which is usually easily cured in people who are not immunocompromised. For example, higher prevalence of colonization of *C. albicans* was reported in young individuals with tongue piercing, in comparison to non-tongue-pierced matched individuals ^6^. Long term untreated infection of Candidiasis in women can block the fallopian tubes hence resulting into infertility among couples. To infect host tissue, the unusual unicellular yeast-like form of *Candida albicans* reacts to environmental cues and switches into an invasive, multicellular filamentous form, a phenomenon called dimorphism ^1^. *Candida albicans* produces a range of extracellular enzymes that facilitate adherence and/or tissues penetration. Phospholipase A, B, C and lysophospholipase may function to damage host cell membranes and facilitate invasion. *C. albicans* produces a range of acid proteinases which have been shown to aid adherence and invasion but which also play an important role in the degradation of the immunoglobulins IgG and IgA. The proteinases have a low pH optimum and this may assist the yeast colonization of the vagina, which is a low pH environment. Haemolysin production by *C. albicans* has also been documented and seems to be important in allowing the yeast access to iron released from ruptured red blood cells ^7^. An important immune evasion tactic of this organism is the ability to bind to platelets via fibrinogen-binding ligands, which results in the fungal cell being surrounded by a cluster of platelets. *Candida albicans* is capable of giving rise to a variety of inter-convertible phenotypes which can be considered as providing an extra dimension to the existing virulence factors associated with this yeast. A number of switching systems have been identified and phenotypic switching may have evolved to compensate for the lack of variation achieved in other organisms that utilize sexual reproduction. Phenotypic switching allows the yeast to exploit micro-niches in the body and alters a variety of factors (example is antifungal drugs resistance, adherence, extracellular enzyme production), in addition to the actual phenotype and so may be considered as the ‘dominant’ or controlling virulence factor.

According to Sheehan *et al.* ^8^ investigation which stated that in terms of tissue colonization and invasion, adherence is the initial step in the process. Once the yeast has adhered, enzymes (phospholipase and proteinase) can facilitate adherence by damaging or degrading cell membranes and extracellular proteins. Hyphae may be produced and penetrate layers of cells using thigmotropism to find the line of least resistance. The passage through cells is undoubtedly aided by the production of extracellular enzymes ^9^. Once endothelial cells are reached, enzymes may assist in the degradation of tissues and allow the yeast to enter the host’s bloodstream, where phenotypic switching or coating with platelets may be used to evade the immune system. While in the bloodstream the haemolysin may function to burst blood cells and release iron, which is essential for growth. Escape from the bloodstream involves adherence to the walls of capillaries and passage across the wall. This work is thus undertaken to evaluate the antimicrobial effectiveness of common disinfectants on *Candida albicans* isolated from five abattoirs displaying tables in Port Harcourt, Nigeria. This is to enable us determine the most potent of the test disinfectants to be used to sanitize these tables before any meat product is displayed for sale to avoid contamination of the meat products.

## Materials and methods

### Test materials and reagents

A selective medium Sabouroud Dextrose Agar (SDA) was used for the cultivation of *Candida albicans*. The following equipments were used-Microscope, Microscopy slides, cover slips, cotton wool, sterile swab sticks and blood serum, safranin, lugos iodine, gram differentials, crystal violet.

### Source of sample

Five different abattoirs (slaughter floor) in an open environment were sampled using sterile swab sticks, within Port Harcourt metropolis, Rivers State Nigeria. The abattoirs include Agip, Woji, Trans Amadi, Rumuokoro and Rumuodara. Samples were collected with sterile cotton swabs moistened with 1ml of 0.1% NaCl peptone solution, from a surface area of preferable 20cm, marked with sterile template and swabbed 10 times from top to bottom ^10^. Then for wet areas dry sterile cotton swabs were used. The swabs were held in sterile forceps and the sampled surfaces were randomly collected every other week for a period of one and a half year starting from January 2005 to June 2006. The samples were taken to the laboratory immediately and cultured.

### Media

Growth media used for enumeration and characterization were selective according to the cultural requirements of fungi. Sarboroud Dextrose Agar (SDA) was used for *Candida albicans* cultivation and the same agar medium for stock culture. The medium was steam sterilised at 121°C for 15 minutes.

### Isolation of the fungi

The spread plate method was used, serial dilutions of the swab sample was made by adding 1.0ml of phosphate buffer into the swab container and mixed thoroughly. From this, serial dilution was carried by adding 9ml of sterile distilled water to give 10 ^-1^, 10 ^-2^, 10 ^-3^, 10 ^-4^ and so on. 0.1ml was plated in triplicate onto sterile selective medium mentioned above. The sets of plates were then incubated at 37°C. Resulting pure colonies were transferred onto SDA agar for subsequent characterization and identification.

### Characterisation and identification

Pure cultures of fungi (yeast) *Candida albicans* isolated were characterized and identified on the basis of their cultural, morphological and biochemical properties and based on their macroscopic appearance on culture medium, and type of asexual spores produced and germ tube test, also identified by reference to Illustrated General of Imperfect Fungi ^11^ and Fungi in Agricultural soil ^12^. The test yeast used is *Candida albicans*.

### Preparation of yeast suspension

Organisms used in this experiments were, *Candida albicans*. *Candida albicans* were grown in Sarboroud Broth overnight. Cultures were centrifuged at 512g (sigma model 3k-1) for 10mm and resulting cell pellets resuspended in 0.1% peptone.

### Preparation of test disinfectants

Disinfectants parachlorometaxylenol (PCMX) (dettol), savlon, purit and Sodium hypochlorite (jik) active were diluted in sterile distilled water prior to use to 10%, 20%, 40% and 70% concentrations these products were obtained from Reckit Benckiser Nigeria Limited Lagos, Nigeria, Johnson & Johnson, Lagos; CAP Plc (Chemical and Allied Products), Lagos respectively and used without further purification. The pH of the mixtures was controlled by the addition of HCL or NaOH as appropriate.

### Suspension test (Traditional Plate Count Method)

Approximately 0.1ml of a yeast suspension approximately 1 × 10 ^9^ bacteria (Cfu/ml) was added to the test disinfectants (10ml), mixed thoroughly and left at room temperature for a specified contact time starting from 0 min, 10 min, 20 min, 30 min, 40 min, 50 min and 60 min. This experiment occupied 21 test tubes each, for the experiment was carried out in triplicate. Following contact an aliquot (1ml) was transferred to universal quenching agent, UQA (9ml of a solution containing 1g peptone, 5g. Tween-80, 1g sodium thiosulphate and 0.7g lecithin 1 ^-1^ of deionised water pH7), for up to 60 min to inactivate the disinfectants. The quenched solution were serially diluted in sterile distilled water and survivors enumerated on SDA Agar (Oxoid) using 0.1ml spread plates. The colonies on the plates were counted after incubation at 37°C for 48hrs. Control test did not contain the disinfectants, but only the serially diluted suspension was plated and counted.

### Composition of disinfectants used

Dettol’s composition include Chlorxylnol B.P.C. 4.8% w/v, Oleum Pini Aromaticum 9.0% v/w Denatured spirits 11.3% w/v, sapo vegetails 5.8% w/v, Saccharum Ustumqs, aqua ad 100vol. Purit contains Chlorhexidine Gluconate B.P. 0.3% w/v and Centrimide B.P. 3.0% w/v. Savlon constitutes n-Propyl alcohol 2.84% m/v, preservative Chlorhexidine gluconate 0.3g and Centrimide 3.0g all in 100ml. Hypochloride contains sodium hypochlorite as an active ingredient.

### Phenol co-efficient test

Rideal-Walker Phenol co-efficient experimental test method for the evaluation of test disinfectants as jointly reported by S. Rideal and J.T.A. Walker in 1903 ^13^ was the first real attempt to evaluate the effectiveness of disinfectants on a qualitative basis. The standard procedure for this test was published by the British Standard Institution.

### The microbial culture for phenol co-efficient testing

The test organism was *Salmonella typhi* NCTC 786 obtainable from the National Collection of typed cultures in London. It was supplied in the freeze-dried form and reactivated by weekly sub-culture on a Rideal-Walker agar slope, incubated at 37°C for 24hrs and then stored at room temperature until the time for another sub-culture. Only cultures that were 22 to 26 hours old and only those from the 3 ^rd^ to the 14 ^th^ subculture were used for the test.

### Disinfectant dilutions

The standard phenol solution contained 1g of phenol in 95, 100, 105, 110 and 115ml of solutions made with sterile distilled water. For the test disinfectants dettol, purit, savlon and jik, four dilutions were made, varied in arithmetic series, changing by the unit of 50; these are 1:200, 1:250, 1:300 and 1:350.

### Procedures

A volume of 5ml of each of the dilutions of test disinfectants and standard phenol was placed in separate sterile test tubes. A 24 hour broth culture of the test organism was also prepared. The dilutions and the culture were placed in a water bath maintained at 17–18°C. When the contents of the tubes and the culture had attained the operational temperature, 0.2ml of the culture was transferred to each of the dilution and shook gently to begin the action of the germicide on the cells. At 2½ minute interval following the incubation of the tubes, a standard loopful of the reaction mixture was transferred to 5ml of sterile nutrient broth (the recovery medium) in a tube. In this way, each reaction mixture was sub-cultured into four separate test tubes of the recovery medium at intervals of 2½, 5, 7½ and 10 minutes respectively. The tubes of recovery medium were incubated at 37°C for 48 hours after which the presence or absence of growth in each tube was recorded.

## Results

**Figure 1.**
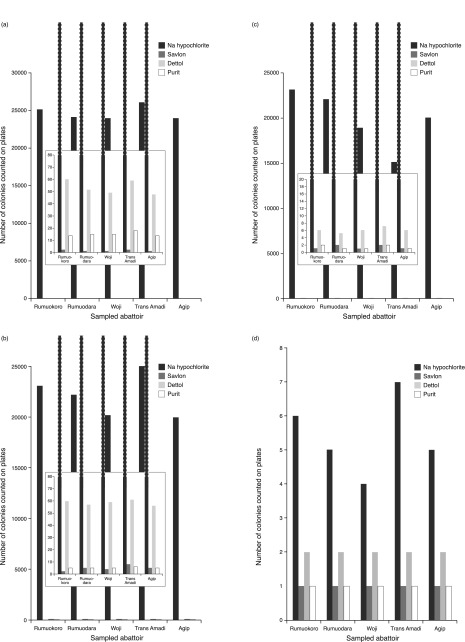
Disinfectant-resistant *Candida* isolates at 10% ( **a**), 20% ( **b**), 40% ( **c**) and 70% ( **d**) concentration.

Figure 1a shows the graph of disinfectants resistant *Candida* isolates at 10% concentration. From this graph it could be deduced that savlon was the most potent disinfectant to *Candida* species, followed by purit, then dettol, while Sodium hypochlorite was the least potent disinfectant to *Candida* isolates. From this result, we can conclude that savlon was the best disinfectant to be used to disinfect *Candida* contaminants on any inanimate objects.

Figure 1b showed the graph of disinfectants resistant *Candida* isolates at 20% concentration. From this graph, it could be deduced that *Candida* species, were more sensitive to both savlon and purit than to dettol and jik (Sodium hypochlorite). *Candida* species were more sensitive to purit and savlon, less sensitive to dettol and least sensitive to Sodium hypochlorite.

## Discussion and conclusion

### Discussion

Laboratory testing of disinfectants was carried out by assessing the sensitivity of various *Candida albicans* strains to biocidal agents using microbial suspension test. Similar observations have been made on bacterial suspensions such as *Staphylococcus aureus*, *Pseudomonas aeruginosa*, *Streptococcus faecalis*, *Proteus mirabilis* and *Escherichia coli* on disinfectants ^14^. Disinfectants used for the test are chlorine based and these included dettol, savlon, purit and jik containing sodium hypochlorite.

From the results of this investigation, it was discovered that *Candida albicans* species were readily isolated from raw meat and from the equipments used in abattoirs. Similar observation has been made by Haniel (1989) ^15^ from his investigation and he stated that undetected *Salmonella* species in instrument or food products such as raw meat could pose a health risk, because they are not heat treated and raw animal tissues may harbor many potentially pathogenic organisms, including bacteria and yeasts e.g. ( *Salmonella*, *Escherichia coli*, *Candida* species) and parasites. For these reasons, the Food and Dietary Association, FDA has promulgated a regulation specifying that meat scraps or other similar animal products are adulterated when they are found upon examination to be contaminated with *Candida* and *Salmonella* microorganisms. According to the investigation of Kirk (2005) ^16^ from Minnesota Department of Agriculture, title 21 code of Federal Regulations (CFR) Part 500.35 stated that not only are the animals consuming the product at risk of infection by organism contained in the raw tissues, but the people handling the product are also at risk. He then stated that adequate heat treatment is the most effective and efficient means of mitigating this risk. Because he reported some processes currently used, such as freezing or freeze drying, do not achieve the same degree of effectiveness as heat treatment. *Candida species* were readily isolated from the tables where meat carcasses are displayed. Similar observation was made by dEnfert and Hube (2007) ^2^ where he stated that these fungi and bacteria are potentially fatal to human especially when ingested and when the immune system is compromised. Ingestion of these yeasts in meat products could be very dangerous or when it has a contact with urinogenital tracts, during defecating or urinating, from contaminated hands and fingers. These fungi cause vomiting and diarrhea, many ailments such as urinary tract infection, even the long term effects in the tracts could result to blockage of fallopian tubes in women and sterility in men. This could be severe in immunocompromised infected people, while healthy people can recover from these infections. Symptoms of candidiasis usually occur within two to ten days of ingesting the yeast (Cotter and Kavanagh, 2000).

*Candida albicans* strains were isolated in number virtually from all the studied samples which are cutting tables. Similar observation was made by Gardner (1993) ^17^, and dEnfert and Hube, (2007) ^2^. The authors worked on the importance of Quality Assurance and Food Safety in Modern Food Production System, they stated that in countries that have implemented a consistent mandatory meat inspection, this harvest food safety procedure and the more and more stringent rules for post-harvest safety measures improving the hygiene standards during slaughter, meat process and distribution have led to a remarkable decline of meat related food-borne disease. And in respect to meat management, it is advised that any animal that arrives at the abattoir should be slaughtered immediately, but animals which have not been slaughtered within 12 hours of the arrival should be fed, and subsequently be given moderate amounts of food at appropriate intervals. Also suitable drinking water should be available to the animals on their arrival and at all times to animals in lairages unless they are to be slaughtered immediately. There has been criticism of the methods of preparation hutching, and killing within some slaughter houses and in particular of the speed with which the slaughter is sometimes conducted ^18– 21^ (also see PETA video taken inside AgriProcessors Inc. in Iowa in 2004. Warning: graphic images). There has also been criticism of the methods of transport of the animals, who are driven for hundreds of miles to slaughter houses in conditions that often result in crush injuries and coma en route, a good condition for microbial contamination and infection ^22, 23^. *Vibrio cholera* the causative agent of cholera, is sensitive to the low pH found in the human stomach, a high infectious dose of 10 ^8^ bacteria is required for the onset of severe cholera; however the infectious dose can drop to 10 ^4^ bacteria in individuals who produce less stomach acid including young children, the elderly and those who take antacids.

From the overall report of this study, dettol at 10% is an appropriate disinfectant or sanitizer that can be used to sanitize the abattoirs cutting tables before any meat products are displayed for sale and should be rinsed off with sterile and clean water after 10 minutes of application. The need for good quality water becomes more and more pressing in the face of the increasing pollution of our water bodies by human activities such as those related to abattoirs services, agriculture and industries. Good water is essential for washing the slaughtered animals, displaying tables, equipments use such as knives, apron, slaughter floor, besides animals themselves need portable water for drinking before slaughter while kept in lairage (see: "Molecule of the Month: Dettol" and "'Dettol Man’ cleans himself to death"). Revankar and Lester (2007) ^24^ stated that overuse of dettol could provide a selective environment in which existing resistant bacteria can grow with little competition. In Nigeria, there is a need for the receiving streams around these abattoirs to be treated because people living in these environments defecate into the water bodies, this act should be discouraged since these are flowing rivers and could serve as sources of drinking and cooking to neighboring villages nearby. This could be the cause of high *Vibrio* isolates in these abattoirs.

This law of meat inspection makes food safer and is the principle of food hygiene being the most important means of protecting consumer against food-borne health risks, in order to reduce food infection. Nevertheless, the receiving streams around these abattoirs should be treated because people living in abattoirs environment defecate inside these water bodies, this act should be discouraged since, they are flowing rivers and could serve as sources of drinking water to neighboring villages where their flow goes. This may be one of the reasons why greater numbers of *Candida* species were isolated from these study abattoirs.

The federal government and local government should be blamed for these vices, because the abattoirs’ chairman, the butchers, the sellers and the inhabitants of these areas have the propensity to live in such a stench environment due to lack of both intuitive and acquired knowledge in boosting the standard of hygiene in abattoirs. Therefore, the government should enforce a legislative law with respect to the standard of hygiene that should be practiced among the abattoirs users, and a circular should be passed round in such a way that everybody should be a participant and there should not be a room for exculpation by any member whatsoever. And anyone that violates this rule should be brought to face the music.

It is advised, due to the overall report of this study, *Candida albicans* is more sensitive to savlon than to other test disinfectants in Port Harcourt abattoirs, hence savlon should be recommended as a potent sanitizer on the abattoirs’ displaying tables and in Nigeria as a whole. Savlon was recommended for destroying *Candida albicans* contaminants within 10 to 20 minutes of exposure to this disinfectant. Phenol co-efficient experimental calculation showed that phenol was less potent than dettol having phenol co-efficient of 7, on *Salmonella* sp. 2 for Dettol on *Pseudomonas* sp. but having 1 on *Pseudomonas* sp. purit, and savlon were more potent than phenol having phenol co-efficient of 1.7, 2 on *Salmonella* sp. and *Pseudomonas* sp. respectively, but equal in phenol co-efficient with phenol on *Staphylococcus* sp. of 1. According to the investigation of De bruin (1976) ^25^ who stated that phenol is generally protoplasmic poison and was the first antiseptic to be employed by Joseph Lister (1912–1927) so it does not make it more potent than the newly produced antiseptics or disinfectants due to the development science and technology. Though, Keswick *et al.* (1985) ^26^ stated that chemical agents used as germicides destroy microbial pathogens using a variety of biochemical mechanisms, including cell membrane.

The results of this study are in agreement with that of Le Chevalier *et al.* (1998) ^27^ which stated that pathogens resistance to chemical disinfectants is typically an inherent characteristic and not one that is acquired or initiated due to exposure to disinfectants. He also stated that mechanisms of resistance of microorganisms have evolved with specialized structures aiding in their survival. Certain *Candida albicans* strains produce a gelatinous material known as proteins to form a biofilm. Biofilm help organisms stick to environmental surfaces and physically protect them from exposure to harmful disinfectants or other detrimental environmental conditions ^28^. This slimy substance can be 100 or more times the mass of the bacterial cells. While the bacteria that initiate biofilm are often not harmful, pathogenic bacteria may also stick to the biofilm and share the protective nature of the environment Hardie *et al.* (2009) ^29^. *Candida* species was very sensitive to most of the tested disinfectants in this study. These sensitivity and resistant attributes exhibited by these microbial isolates may be due to some of the innate characteristics stated above. The high incidence of water and food borne sanitation diseases is one of the major problems confronting our country, Nigeria, due to unhygienic abattoir environments.

In conclusion, the result of this research has shown that for our meat products to be free from Candida contaminants, savlon is an effective sanitizer on abattoirs cutting tables and should be applied at 10% concentration for 10 minutes and then rinsed off with sterile water before meat products are displayed for sale. This automatically reduces the yeast load on these objects.

The following recommendations are made as follows:

The federal government should set up a group of Environmental Health workers which will comprise of Environmental Microbiologists, Veterinary Doctors, Medical Doctors and Medical Laboratory Scientists that would play a significant role in the promotion of the health of the people. Adequate sanitation and safe water are fundamental prerequisites for Nigeria abattoirs as a whole for better health, otherwise other programs may not succeed and there can be lasting improvement of public health. These should be vigilantly implemented by these personnel listed above including sanitary engineers and inspectors, and should be directly involved in this exercise.

It was discovered from this study that every abattoir has a receiving stream and this stream or river could serve as a source of drinking water to some rural dwellers around the vicinity, hence the butchers and other inhabitants in abattoirs defecate into this same river. The government should abrogate these vices by providing good toilet facilities for them, for water quality control, proper excreta disposal and Food sanitary inspection should be practiced (Food Law internet Project (FLIP) 2000. Food safety Administration. Authorized information provided to the WHO. University of Reading UK, Michigan State University, U.S.A) ^30^.
