# Supplementary material for: The efficacy of disinfectants on abattoirs’ Candida albicans isolates in Niger Delta region
Source: F1000Res. 2012 Oct 1;1:20. [Version 1] doi: 10.12688/f1000research.1-20.v1 (PMC3799549; doi:10.12688/f1000research.1-20.v1)
Supplement: Peer review history

# Peer review: Referee response for version 1

**Published:** 2013-05-08

**Authors:** Astrid Mayr

**Affiliations:** 1 Department of Hygiene, Microbiology and Social Medicine, Division of Hygiene and Medical Microbiology, Medical University Innsbruck, Innsbruck, Austria

I think this is an interesting manuscript which provides good information about basic hygienic issues, like disinfection and prevention of infection.

I have read this submission. I believe that I have an appropriate level of expertise to confirm that it is of an acceptable scientific standard.

# Peer review: Referee response for version 1

**Published:** 2012-11-20

**Authors:** Ifeoma Enweani

**Affiliations:** 1 Department of Medical Laboratory Science, Faculty of Health Sciences and Technology, Nnamdi Azikiwe University, Akwa, Nigeria

I have read this submission. I believe that I have an appropriate level of expertise to confirm that it is of an acceptable scientific standard.
